# Completion Pancreaticoduodenectomy for a Second Primary Pancreatic Cancer: A Case Report

**DOI:** 10.1089/crpc.2016.0010

**Published:** 2016-06-01

**Authors:** Jeffrey M. Hardacre

**Affiliations:** Department of Surgery, University Hospitals Case Medical Center, Cleveland, Ohio.

**Keywords:** completion pancreatectomy, recurrent pancreatic cancer

## Abstract

**Background:** Recurrent pancreatic cancer may represent a true local recurrence or a second intrapancreatic primary. Resection of recurrent pancreatic cancer is uncommon.

**Case Presentation:** A 68-year-old woman underwent a distal pancreatectomy/splenectomy for pancreatic adenocarcinoma after presenting with acute pancreatitis. She received 6 months of adjuvant gemcitabine. Nearly 5 years later, she presented with acute pancreatitis. Endoscopic ultrasonography suggested malignant degeneration of an uncinate intraductal papillary mucinous neoplasm (IPMN), but the cytology was negative. She subsequently underwent a completion pancreaticoduodenectomy for what proved to be a second pancreatic adenocarcinoma.

**Conclusion:** Although uncommon, repeat resection for a second pancreatic cancer may be appropriate in select patients. Careful attention to the remnant pancreas must be maintained on surveillance imaging.

## Introduction

Recurrence after resection of pancreatic cancer may manifest as a local recurrence, peritoneal recurrence, or distant recurrence. The latter two are not amenable to repeat resection. Local recurrence may represent a recurrence in the original resection bed, an intrapancreatic recurrence, or a second primary pancreatic cancer. Differentiating between an intrapancreatic recurrence and a second primary pancreatic cancer can be difficult. Factors to consider include the location and timing of the recurrence.

Here, we present a case of a second primary pancreatic cancer resected nearly 5 years after distal pancreatectomy.

## Presentation of Case

**FIG. 1.**
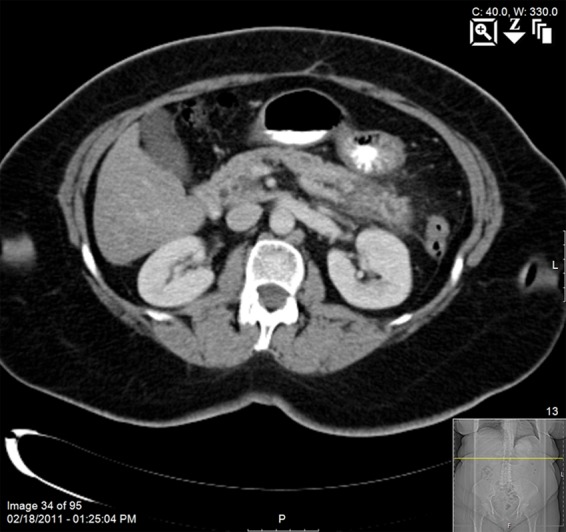
CT scan at initial presentation with acute pancreatitis in 2011.

The patient is a 68-year-old woman who presented with acute pancreatitis in February 2011. CT (Fig. 1) and magnetic resonance imaging at the time showed inflammatory change around the body/tail of the gland, ectasia with side-branch sacculations in the uncinate process, and a pancreatic duct stricture in the body/tail. Endoscopic ultrasound (EUS) and endoscopic retrograde cholangiopancreatography described clear, thin fluid flowing from a nonpatulous papilla. Saccular changes and cystic spaces that connected to the main pancreatic duct were described in the head of the gland. A stricture was seen in the pancreatic duct in the body/tail without an obvious mass. Fine needle aspiration (FNA) at the site of the stricture yielded malignant cells derived from adenocarcinoma. The patient completed staging for her newfound pancreatic cancer and had no evidence of disseminated disease. The clinical impression was that she had a pancreatic body/tail cancer that caused her pancreatitis and that the changes in the head/uncinated lesion reflected either a branch-duct intraductal papillary mucinous neoplasm (IPMN) or ectatic side ducts.

She underwent distal pancreatectomy/splenectomy in March 2011 for what proved to be a moderately differentiated, T1N0 pancreatic ductal adenocarcinoma with the margin free of invasive cancer or high-grade dysplasia. PanIn 1B was noted at the margin. She completed 6 months of adjuvant gemcitabine.

**FIG. 2.**
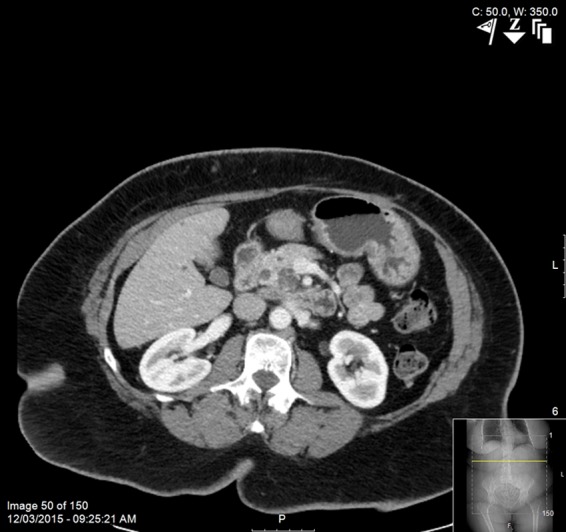
CT scan in 2015 showing stable uncinate cystic lesion and no associated mass.

Under surveillance, she underwent 10 abdominal CTs after resection through March of 2015, which showed no evidence of recurrence and no change in the cystic area in the uncinate process of the pancreas. In late November 2015, she experienced some mild abdominal pain and bloating. A CT (Fig. 2) showed an essentially stable cystic lesion in the uncinate with no associated mass. In late December 2015, she presented with acute pancreatitis with stranding around the head and uncinate on noncontrasted imaging. An EUS in January 2016 found a 2.9 cm hypoechoic mass with irregular borders. There was mild prominence of the main pancreatic duct and side branches. An adequate FNA specimen showed no evidence of malignant cells.

Based on this patient's personal history of pancreatic cancer, based on her presentation with pancreatitis, and based on her imaging/EUS findings, completion pancreaticoduodenectomy was recommended for a working diagnosis of branch-duct IPMN, possibly invasive. The patient underwent a completion pancreaticoduodenectomy for what proved to be a moderately differentiated, T3N0 pancreatic ductal adenocarcinoma with negative margins. PanIn 3 was seen elsewhere in the specimen. The morphology of this cancer was similar to that of her initial resection specimen. Adjuvant chemotherapy has been started.

## Discussion

After resection for pancreatic cancer, the majority of patients recur and ultimately die of their disease. Peritoneal and distant recurrences are treated with systemic therapy. Local recurrence is usually treated with systemic therapy; however, radiation therapy may be considered in select circumstances. Rarely is repeat resection done for locally recurrent pancreatic cancer.^1,2^

Locally recurrent pancreatic cancer may appear in the original resection bed or in the remnant pancreas. If in the remnant pancreas, the tumor may be a true recurrence or a second pancreatic primary. Distinguishing between a true recurrence and a second primary is done clinically on the basis of the site of recurrence, that is, is the recurrence at or near the original plane of transection, and the timing of the recurrence. The patient presented in this report was felt to have a second pancreatic primary as her recurrence was well away from the transection plane at the time of distal pancreatectomy and because the diagnosis was made nearly 5 years after her initial resection.

Intrapancreatic recurrence of pancreatic cancer could be explained by a close margin at the time of original resection or by the finding of high-grade dyplasia (PanIn 3) at the margin. A second pancreatic primary could be explained by the findings of Tryka and Brooks as well as Launois et al.^3,4^ Both groups found multifocal carcinoma in total pancreatectomy specimens. Such multifocality would likely present earlier than 5 years after a partial resection. Our patient's second pancreatic primary is more likely explained by the findings of Andea et al., who found that in 40% of pancreatic resection specimens for cancer, there was separately identified PanIn 3.^5^ Given the fact that this patient had PanIn 1B at her original transection margin and that she had PanIn 3 seen elsewhere in her second resection specimen, we assume she had progression of a dysplastic lesion in her pancreatic duct that led to her second primary. Our working diagnosis going into the second operation was that she had a branch-duct IPMN, possibly with malignant degeneration. This turned out to be incorrect based on final pathology as reviewed by two experienced gastrointestional pathologists. They did not see any evidence that the second cancer arose in the setting of an IPMN.

As exemplified in this case report, second primary pancreatic cancers can appear. Given the propensity for PanIn lesions to be found along with invasive cancers in resection specimens, attention must be paid to the remnant pancreas on surveillance imaging. In select circumstances, repeat resection should be considered.

## Abbreviations Used

CT: computed tomography
EUS: endoscopic ultrasound
FNA: fine needle aspiration
IPMN: intraductal papillary mucinous neoplasm

## References

[1] Kleeff J, Reiser C, Hinz U, et al. Surgery for recurrent pancreatic ductal adenocarcinoma. Ann Surg. 2007;245:566–572 17414605 10.1097/01.sla.0000245845.06772.7d PMC1877037

[2] Miyazaki M, Yoshitomi H, Shimizu H, et al. Repeat pancreatectomy for pancreatic ductal cancer recurrence in the remnant pancreas after initial pancreatectomy–is it worthwhile? Surgery. 2014;155:58–66 24238124 10.1016/j.surg.2013.06.050

[3] Tryka AF, Brooks JR Histopathology in the evaluation of total pancreatectomy for ductal carcinoma. Ann Surg. 1979;190:373–381 485612 10.1097/00000658-197909000-00013 PMC1344674

[4] Launois B, Franci J, Bardaxoglou E, et al. Total pancreatectomy for ductal adenocarcinoma of the pancreas with special reference to resection of the portal vein and multicentric cancer. World J Surg. 1993;17:122–126 8383381 10.1007/BF01655724

[5] Andea A, Sarkar F, Adsay VN Clinicopathological correlates of pancreatic intraepithelial neoplasia–a comparative analysis of 82 cases with and 152 cases without pancreatic ductal adenocarcinoma. Mod Path. 2003;16:996–1006 14559982 10.1097/01.MP.0000087422.24733.62

